# Integrating inverse reinforcement learning into data-driven mechanistic computational models: a novel paradigm to decode cancer cell heterogeneity

**DOI:** 10.3389/fsysb.2024.1333760

**Published:** 2024-03-08

**Authors:** Patrick C. Kinnunen, Kenneth K. Y. Ho, Siddhartha Srivastava, Chengyang Huang, Wanggang Shen, Krishna Garikipati, Gary D. Luker, Nikola Banovic, Xun Huan, Jennifer J. Linderman, Kathryn E. Luker

**Affiliations:** ^1^ Departments of Chemical Engineering, University of Michigan, Ann Arbor, MI, United States; ^2^ Radiology, University of Michigan, Ann Arbor, MI, United States; ^3^ Mechanical Engineering, University of Michigan, Ann Arbor, MI, United States; ^4^ Michigan Institute for Computational Discovery and Engineering, University of Michigan, Ann Arbor, MI, United States; ^5^ Mathematics, University of Michigan, Ann Arbor, MI, United States; ^6^ Biomedical Engineering, University of Michigan, Ann Arbor, MI, United States; ^7^ Electrical Engineering and Computer Science, University of Michigan, Ann Arbor, MI, United States; ^8^ Biointerfaces Institute, University of Michigan, Ann Arbor, MI, United States

**Keywords:** inverse reinforcment learning, mechanistic modeling, machine learning, cellular heterogeneity, live-cell microscopy

## Abstract

Cellular heterogeneity is a ubiquitous aspect of biology and a major obstacle to successful cancer treatment. Several techniques have emerged to quantify heterogeneity in live cells along axes including cellular migration, morphology, growth, and signaling. Crucially, these studies reveal that cellular heterogeneity is not a result of randomness or a failure in cellular control systems, but instead is a predictable aspect of multicellular systems. We hypothesize that individual cells in complex tissues can behave as reward-maximizing agents and that differences in reward perception can explain heterogeneity. In this perspective, we introduce inverse reinforcement learning as a novel approach for analyzing cellular heterogeneity. We briefly detail experimental approaches for measuring cellular heterogeneity over time and how these experiments can generate datasets consisting of cellular states and actions. Next, we show how inverse reinforcement learning can be applied to these datasets to infer how individual cells choose different actions based on heterogeneous states. Finally, we introduce potential applications of inverse reinforcement learning to three cell biology problems. Overall, we expect inverse reinforcement learning to reveal why cells behave heterogeneously and enable identification of novel treatments based on this new understanding.

## Introduction

There is an enigma at the heart of mammalian biology. Seemingly identical cells in a population exhibit distinct responses to the same environmental cues. *Consequences* of heterogeneity are readily apparent in normal biology and diseases such as cancer: specialized behaviors of cells, drug resistance, and fatal metastases. Mechanisms *causing* heterogeneity remain a mystery, impeding efforts to shift cell behaviors to prevent or cure disease.

The prevailing dogma is that heterogeneity among cancer cells arises randomly, generating “greedy individuals” that compete for growth factors and optimal environments. However, recent data suggest that cancer cells function cooperatively as a tissue-like entity, and work by our group and others demonstrate that single-cell differences in signaling and function among cancer cells can arise predictably with consistent variations across a population as a whole (Spencer et al., 2009; Overton et al., 2014; Spinosa et al., 2019; Spinosa et al., 2020; Zhan et al., 2020; Kinnunen et al., 2022). These observations imply that tumor progression benefits from or even requires interactions among distinct subgroups of cells (Marusyk et al., 2014). The idea that single, heterogeneous cancer cells work collectively within a constrained range of variability to drive population-level outputs in tumor progression is a concept that may revolutionize how we approach cancer biology and therapy.

To decipher mechanisms regulating single-cell heterogeneity and cooperative interactions among cells, we propose that the field adopt a conceptual approach that integrates: (1) high-dimensional single-cell data, (2) mechanistic modeling, and (3) inverse reinforcement learning (IRL). While typically used to imitate (Abbeel and Ng, 2004) or simulate (Banovic et al., 2016) human behavior, IRL is an artificial intelligence (AI) method that can interpret responses of single cells to multiple stimuli as a decision-making *policy* that is motivated by maximizing a *reward*. Key IRL terms, with application to cancer, are defined in Box 1. In the context of cancer, rewards exist at both single cancer cell and multicellular tumor-microenvironmental scales. For cancer cells positioned in nutrient-rich environments, a reward may be activation of signaling pathways that drive metabolic or cytoskeletal adaptations necessary for proliferation and invasion. Treatment with radiation or chemotherapy leads to rewards related to single and tumor-wide behaviors that promote survival (Shaffer et al., 2017). Single cancer cells may upregulate drug efflux transporters and DNA damage repair processes to resist therapy, while soluble and contact-mediated interactions among cancer and benign stromal cells promote survival of the tumor overall (Lim et al., 2011; Li et al., 2021; Xiao et al., 2021). Tumor-wide cellular and metabolic interactions generate immunosuppressive environments that restrain and exclude anti-cancer immune responses (DePeaux and Delgoffe, 2021; Luby and Alves-Guerra, 2021; Arner and Rathmell, 2023). These examples capture only a subset of the many possible reward-induced “decisions” cancer cells make that may support heterogeneity and drive tumor growth and metastasis.

**Box 1:** Key terms in IRL with examples from cancer biology

Term	IRL Definition	Cancer biology example(s)
Agent	An autonomous entity that takes actions in a state-dependent manner to maximize some unknown reward	Cancer cell, stromal cell
State (S)	Variables defining the measurable or model-inferable properties of the agent	Size; location; level of activation of a signaling pathway; readiness to divide; cancer stemness
Action (A)	Performed by agents to transition between states	Moving up a chemical gradient; not moving; cell division; apoptosis; new activation of a signaling pathway
Reward R (S,A)	Benefit that the agent obtains from the environment by taking a particular action when in a particular state. In IRL, the reward is unknown *a priori* and is inferred from observed agent behaviors	High rewards under proliferation or invasion; survival during drug treatment; generation of immunosuppressive environment
Policy	A probabilistic or deterministic mapping from current state to immediate action. IRL assumes that the agent performs the optimal policy maximizing the expected cumulative (unknown) reward over some time horizon	A cancer cell may follow a policy of spending resources to send signals to neighboring stromal cells when it is surrounded by more stromal cells (state), causing them to alter their metabolism to support the cancer cell
Environment	Set of inputs sensed by the agent	Extracellular surroundings, including the presence and concentration of soluble signaling molecules and neighboring cells. Mechanical environment (stiffness) of surroundings

**FIGURE 1:**
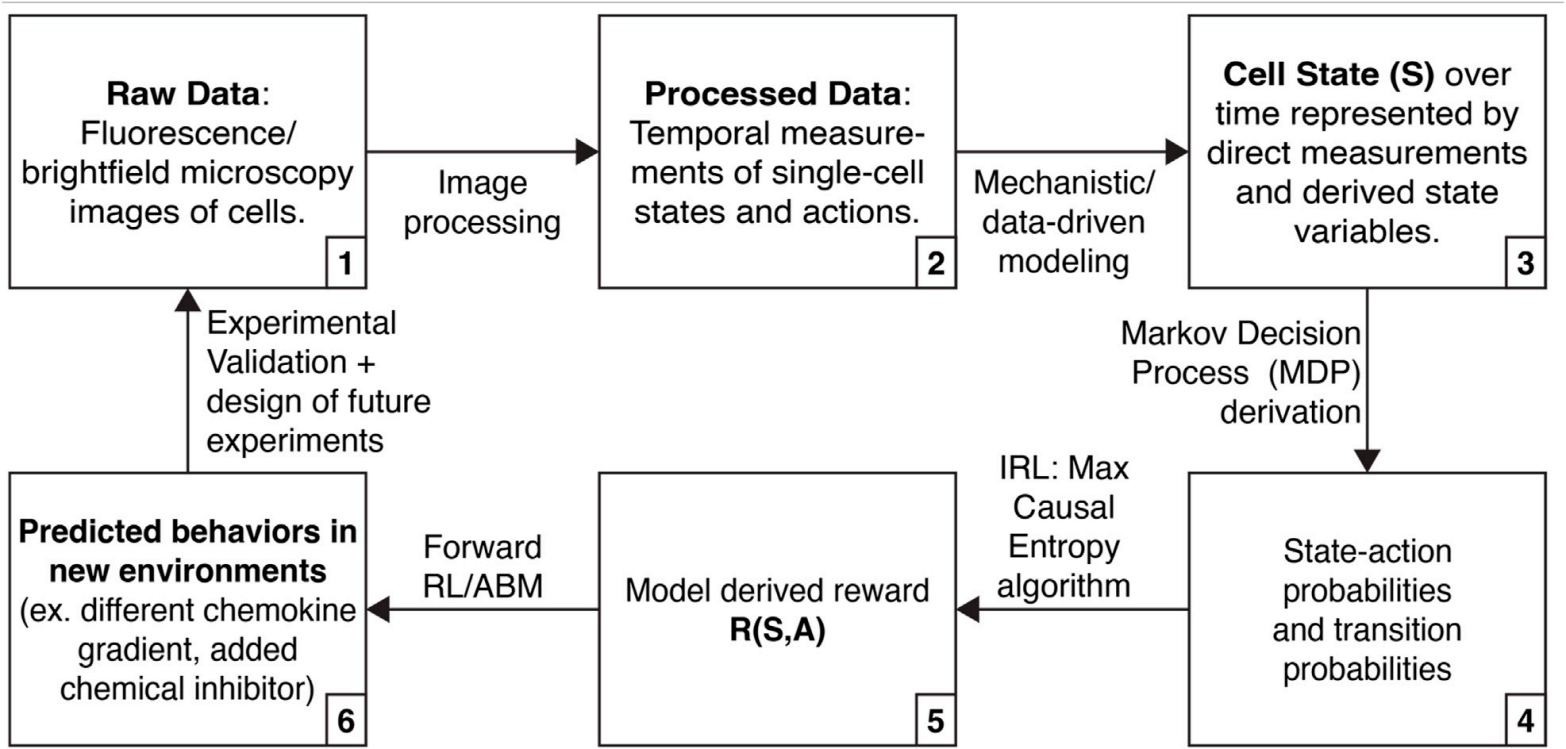
Approach that integrates high-dimensional single cell data, mechanistic modeling, and inverse reinforcement learning (IRL) to learn about cell decision-making.

We describe below how high dimensional single-cell data, mechanistic modeling, and IRL might be integrated to discover molecular processes underlying “decision-making” by single cells and their “motivations” for acting competitively or collaboratively in cancer (Figure 1). By basing IRL findings on single cell data and mechanistic models, we can ensure that the approach yields biologically realistic hypotheses (for example, predicted behaviors in new environments, including reproducing heterogeneity in a population or evading drug treatment).

### Live cell imaging measures heterogeneous cell states and actions

Live-cell microscopy with advanced image processing methods can track and analyze single cells over space and time, measuring cellular phenotypes such as movement, division, proliferation, and death (Figure 1, steps 1 and 2). Stimuli can be applied to cells to measure the response of each cell, and multiple stimuli can be applied successively to determine how various inputs reinforce or counter outputs such as cell signaling and movement. Live-cell microscopy has revealed previously unexamined dimensions of cellular heterogeneity, including morphology (Gordonov et al., 2016), engulfment (Chu et al., 2020), and migratory capacity (Ferreira et al., 2022). Combining live-cell microscopy with a growing array of optical imaging reporters vastly expands the number of measurable phenotypes per cell and dynamic responses of cells over time. As examples, investigators have used multiplexed fluorescent reporters of cell cycle phases, DNA damage, cell signaling pathways, or protein stability/degradation (Sakaue-Sawano et al., 2008; Regot et al., 2014; Spinosa et al., 2019; Suski et al., 2022; Abd El-Hafeez et al., 2023). Dynamic imaging studies generate large datasets by collecting information from thousands of cells over hours to days.

The application of live-cell fluorescence microscopy to cell biology has revealed two key principles. First, even genetically identical cells respond heterogeneously to identical stimuli. There are numerous examples of this heterogeneity in both continuous and discrete cell actions. For example, the Akt, ERK, and p38 kinase pathways display a continuum of signaling activities in response to chemokine stimulation (Kinnunen et al., 2022). Isogenic cells display a heterogeneous spectrum of chemotactic capacities under identical gradients (Ho et al., 2023). Heterogeneity is also present in cellular decisions relating to actions like cell death and cell-cycle progression. Imaging reporters for these processes have revealed intercellular variations in dynamics of cell division, inheritance of cell *states*, and responses to interventions such as chemotherapy drugs (Laughney et al., 2014; Kukhtevich et al., 2022; Arora et al., 2023). The second principle of cellular heterogeneity is that cellular behaviors are influenced by cell *state*, which is set by past stimuli. We and others have used imaging reporters to detect “memory” of past stimuli, responses to targeted therapy, and how oscillations in kinase activity can control single cell decisions regulating transcription, chemotaxis, and apoptosis (Tomida et al., 2015; Hiratsuka et al., 2020; Wang et al., 2022; Heaton et al., 2023; Ho et al., 2023). Heterogeneity has been observed even in more complex environments, including in living tissues and organoids (Hiratsuka et al., 2015; de Witte et al., 2020; Ponsioen et al., 2021). Hence, heterogeneity in cell state appears to be a fundamental property of collections of cells.

Heterogeneity enables at least two emergent behaviors in cancer cells: cooperation and bet-hedging. Cooperation enables cells to specialize to create an overall more oncogenic environment. For example, cancer cells can exploit metabolic byproducts from the microenvironment (Richardson et al., 2018; Zhu et al., 2020), and chemokine-expressing metastatic cancer cells can create a favorable environment for non-expressing cells (Shahriari et al., 2017), which would otherwise die. We can think of the cells that rely on byproducts from other cells as selfish exploiters. Bet-hedging refers to the adoption of phenotypes that are suboptimal in the current environment but may be better suited to potential future environments, such as after the application of a cytotoxic drug (Sharma et al., 2010). Understanding how cancer cells collaborate, and when selfish cancer stem cells emerge, could enable the identification of novel cancer targets.

To work with IRL, we envision that live-cell, fluorescence microscopy combined with automated image processing will provide large data sets of cellular behaviors (Moen et al., 2019; Tian et al., 2020). These datasets can include multiple cell types, complex environments, and the addition of multiple exogenous stimuli (Zhang et al., 2019; Buschhaus et al., 2020; Ho et al., 2023). Such datasets can then be converted into sets of single-cell states and actions, a requirement for IRL. Our current microscopy datasets contain ∼100,000 such data points (state-action pairs), and we can combine data from multiple experiments, providing ample data for training IRL algorithms. Furthermore, innovations in live-cell microscopy and fluorescent reporter design will continue to expand the cell states and actions we can measure. IRL might also be combined with other sources of data that collect time-series data consisting of cell states and actions. However, IRL cannot be performed using only single-cell endpoint measurements, such as flow cytometry or single-cell (spatial) transcriptomics, because they do not provide time series data. Endpoint measurements that can be linked to the states and actions of a specific cell, such as cyclic immunofluorescence, could be used to associate specific behaviors with a wider range of endpoint measurements than can be measured in living cells.

### Physically-based mechanistic models ground IRL findings in reality

Predictions about the drivers of cell behavior need to be placed in a readily understandable, real-world framework for cell signaling and function: physically-based mechanistic models. Systems biologists have already created a broad corpus of knowledge about heterogeneity in single- and multicellular behavior and regulation. For example, mechanistic models in cancer have been developed for multiple signaling pathways, tissue formation, cell migration, and drug treatment, primarily by combining knowledge of biology with principles from biochemistry, biophysics, and engineering, e.g., diffusion and convection, mechanics, and biochemical reaction networks (Spinosa et al., 2020; Kinnunen et al., 2022; Menezes et al., 2022). Such models may include both deterministic and stochastic elements. An emerging data-driven approach for modeling is system inference. For example, Variational System Identification (VSI) techniques allow estimation of the parametric form of the governing partial differential equations–such as reaction-diffusion and phase field models–that may underlie cell migration and signaling, directly from experimental data (Wang et al., 2019; Wang et al., 2021; Ho et al., 2023; Kinnunen et al., 2023).

An important class of models for our discussion is agent-based models (ABMs). In the current context, the *agents* in the models are individual cells, and they behave and interact in their environment according to probabilistic rules. In particular, and relevant to our IRL discussion, we describe the behavior of agents in an ABM through a Markov Decision Process (MDP), a mathematical framework where cell-agents decide their actions from their current states motivated by gaining higher rewards. ABMs model cellular heterogeneity by explicitly representing cell state, placing heterogeneous cells in a varied environment, and following the state changes and actions taken by individual cells over time as the simulation proceeds. There is now a fairly long history of ABMs in biology with rules informed by our knowledge of biology and also, more recently, by machine learning (Norton et al., 2017; Rikard et al., 2019; Hult et al., 2021; Sivakumar et al., 2022). Yet deducing a rule, for example, that cells are likely to move in a certain way in a certain gradient, does not tell us if or why this *action* supports heterogeneity and ultimately cancer survival. This is a difficult problem because the final result (cancer survival, say) is likely many steps removed from any individual cell’s action. For this, we can turn to IRL to determine the *rewards* that drive the *policies* the cells follow.

We envision using mechanistic modeling to improve the interpretability of IRL inference in three ways (Figure 1, steps 3, 4, and 6). First, mechanistic modeling can expand the number of cell states we can use for IRL. Many cell states do not have associated live-cell reporters, and there are limitations on the number of fluorescence reporters that can be simultaneously measured. However, we can fit data to mechanistic models, elucidating additional states (Yao et al., 2016; Spinosa et al., 2020). Second, mechanistic modeling can identify physical limits in cellular actions or state transitions. For instance, previous work has derived physical limits on a cell’s ability to sense a chemical gradient (Mugler et al., 2016). By incorporating these limits into measured state-action transitions, we can prevent IRL from needlessly exploring solutions that are physically inadmissible. Finally, we can use IRL in combination with ABMs to simulate cells following the inferred rewards with controlled perturbations, yielding actionable hypotheses and guiding the design of future experiments (Huan and Marzouk, 2013; Shen and Huan, 2023).

### IRL uncovers cell- and tumor-level “motivations” from observed cell states and actions

Uncovering the underlying incentive mechanism in a complex decision-making system is a formidable task, especially when the system is stochastic and its constituent agents possess substantial heterogeneity. IRL is a powerful tool that harnesses agent-scale data to infer the unknown incentive mechanisms governing the behavior of individual agents. IRL differs from the more commonly used reinforcement learning (RL): in RL (or forward RL) an agent learns a good policy for taking actions from trial and error based on a given (known) reward function; in IRL one tries to discover a reward function based on the behavior of an agent that follows an optimal policy in its environment.

In the IRL framework, we model a cancer cell as a decision-making agent under the mathematical formalism of an MDP (Bellman, 1957). This approach is rooted in the assumption that the agent is a rational actor, and the observed data reflect the agent choosing the optimal state-dependent action to maximize its expected cumulative reward while navigating the constraints of its environment. In other words, the agent is assumed to adhere to an optimal policy for some unknown, underlying reward mechanism. For example, we know that only a small subpopulation of cancer cells in a tumor are metastatic (Luzzi et al., 1998). Using IRL, and assuming that these cells are maximizing an unknown reward, might reveal that metastatic cells undergo a set of specific states prior to metastasis, where migration is highly rewarded. Meanwhile, other nonmetastatic cells do not pass through these states (Marusyk et al., 2014). Furthermore, by comparing the magnitude of the rewards accumulated at each step on the path to metastasis, we could identify the steps taken by metastatic cells that are most important to target therapeutically. IRL provides the mathematical and computational tools to systematically identify other cases where individual cells may adopt seemingly suboptimal phenotypes in order to optimize tumor growth.

In the IRL framework, cells and their surrounding environment (e.g., a neighborhood consisting of various other cells, soluble factors, and mechanical properties) are represented by a set of states. The framework also specifies a set of actions that a cell can take in each of those states (e.g., movement, division, secretion). The cell transitions from state to state appear stochastic for two reasons. First, cell actions can change the environment; for example, the secretion of a cytokine will change the local concentration. Second, cells do not have full control over their environment, and some changes in the environment happen irrespective of cell actions. For instance, a moving cell may intend to move to a region of lower cell density, but since other cells are also moving, it may end up in a region of similar or even higher density. Cells perform actions according to a policy that maximizes a reward function the cell receives after reaching a new state for each action. IRL models cell behavior as a sequence of actions the cell performs as it moves from state to state until reaching some final goal state, such as continuing to proliferate after exposure to a chemotherapeutic drug.

IRL is a method for estimating the rewards of an MDP (Figure 1, steps 3–5). To perform IRL, state-action probabilities are calculated. Here, we envision state-action probabilities being determined both from measured data and augmentation of measurements using mechanistic and data-driven modeling. Next, we parameterize the reward function and use the MaxCausalEntropy algorithm to identify the most likely rewards for each state and action (Ziebart et al., 2010). MaxCausalEntropy is particularly well suited for modeling cellular behaviors because it explicitly models the connection between cell state and cell action, which we assume are connected by (currently incompletely understood) physical and chemical laws.

With an MDP and rewards in hand, we can formulate and test critical hypotheses in cancer biology. We can test whether individual cells in new conditions are behaving consistently with the model, or if they represent outliers displaying new behavior; in other words, how heterogeneous, and in what ways, is the new population? We can calculate the probabilities that cells will exist in particular states, or take particular sequences of actions, to better understand the scope of cell behavior. We can simulate populations of cells under different situations, i.e., make predictions that can then be tested in experiments (Figure 1, step 6). Finally, we can identify a final state of interest (for instance, metastatic or drug resistant cells) and identify the states and actions most likely to lead to that state. These latter examples highlight the ability of the model to enable us to develop targeted interventions to control the behavior of cells.

IRL has had remarkable success in various fields, including human behavior modeling (Antar et al., 2022) and robotics (Finn et al., 2016), but has only recently been applied to biology. IRL was used to understand the clonal evolution of tumors (Kalantari et al., 2020) and mimic the behavior of physicians making cancer treatment decisions (Imani and Braga-Neto, 2019). Two papers apply IRL to study the migration behavior of roundworms (Yamaguchi et al., 2018) and mice (Ashwood et al., 2022), which are particularly relevant for our application. Yamaguchi et al. used IRL to study thermotaxis in roundworms (Yamaguchi et al., 2018). They tracked roundworm migration in a thermal gradient using recordings and automated video analysis, which generated hundreds of single-worm trajectories. They modeled the worm state based on the current temperature and the current temperature gradient. IRL revealed different migration strategies for worms grown in different conditions, which recapitulated prior knowledge about worm thermotaxis. Ashwood et al. applied IRL to mice navigating a maze (Rosenberg et al., 2021; Ashwood et al., 2022). They also used video recordings as a data source and were able to identify different time-varying rewards for water-restricted and -unrestricted mice. The data used in these studies are structurally very similar to the data collected from live-cell microscopy, which suggests that similar techniques may be effective.

### Challenges in applying IRL to cellular behaviors

Despite the effectiveness of IRL in various fields, it comes with significant challenges and limitations. First, IRL is inherently ill-posed since many reward functions exist that can explain the demonstrated trajectories equally well, which can lead to overfitting. Moreover, the ill-posedness can be exacerbated by incomplete or imperfect knowledge about the environmental dynamics and where an explicit, analytical form of the state transition function is unavailable, as in many biological scenarios. These challenges emphasize the need to embed IRL within an experimental framework where inferred rewards can be tested using new experiments incorporating genetic, chemical, or environmental perturbations. Second, IRL may infer rewards that do not make physical sense–for instance, predicting cell division more quickly than cells could possibly divide–or are difficult to interpret. Thirdly, IRL faces challenges related to computational complexity and sample size requirements, both of which usually increase with the dimensionality of the state-action space. Meanwhile, as the problem size increases, more diverse examples of behavior are needed to maintain sufficient coverage in the training data. This need highlights another challenge: generalizability. The difficulty lies in accurately extrapolating to unobserved spaces using data that often covers only a fraction of the total space. Relying solely on observations to generalize to state and action regions beyond training samples becomes especially difficult in high-dimensional settings, compounded when training data are limited and noisy. To help resolve these problems, we emphasize that combining IRL with more traditional biochemical and biophysical modeling will ensure that the learned rewards are physically meaningful and interpretable. An example of this approach recently developed by our team is Fokker-Planck-based IRL (FP-IRL), which we will elaborate below.

### Toward integrating IRL, mechanistic models, and single-cell biology: three potential applications

**FIGURE 2:**
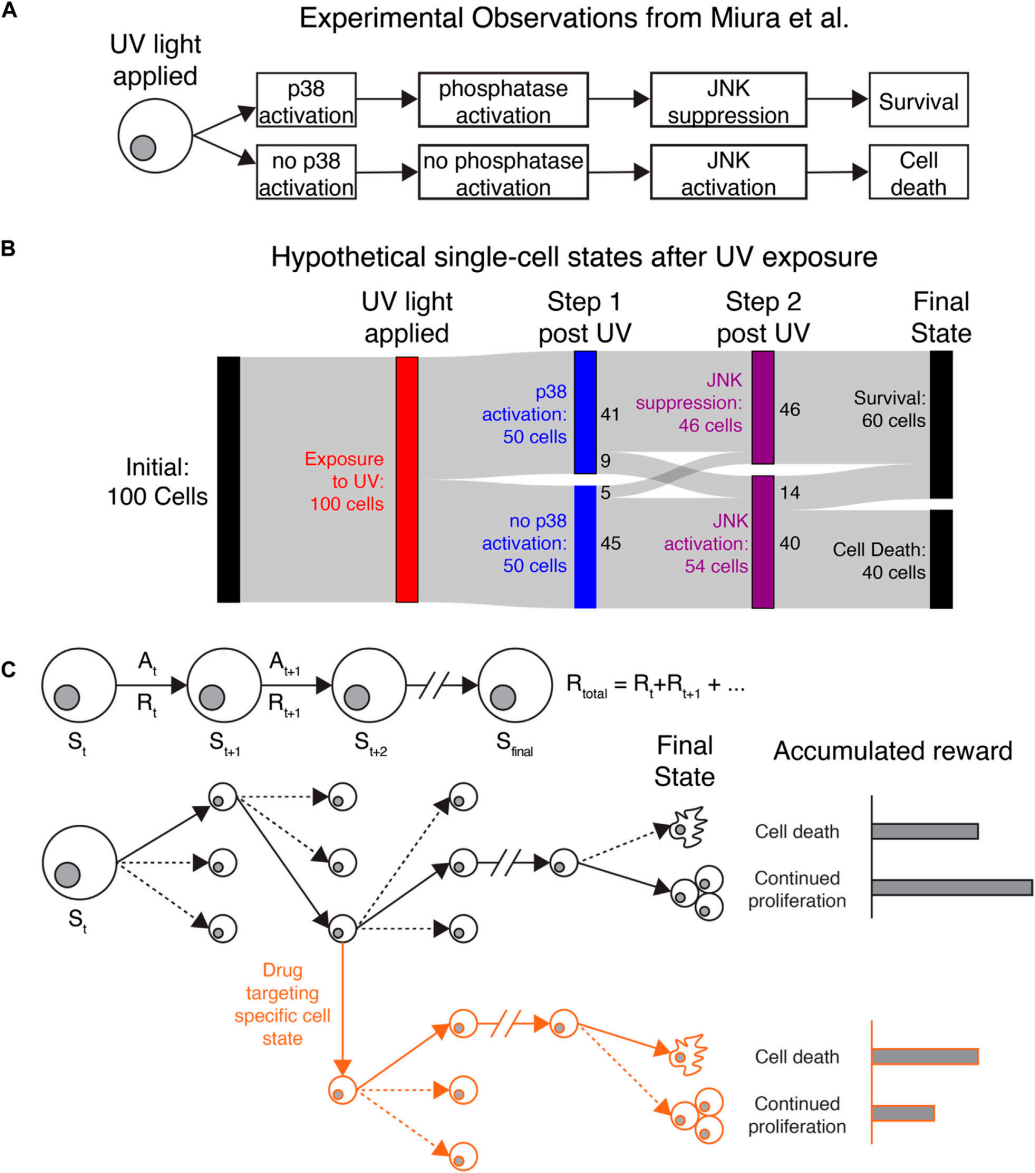
Applying IRL to understand heterogeneous single-cell behaviors **(A)** Original observations from Miura et al. demonstrating that stochastic cell death after UV exposure is due to differences in p38 activation, phosphatase activation, and JNK activation. **(B)** Sankey diagram showing how IRL could be used to study hypothetical data generated based on the observations of Miura et al. Cell states and actions can identify states that affect cell death or continued proliferation after exposure to UV light. Colored bars show different cell states, while the gray bands show how many cells transition between each state. Here, a hypothetical population of 100 cells is uniformly exposed to UV radiation (red). Immediately after radiation, cells either activate or do not activate the protein kinase p38 (blue). Most p38-active cells then suppress the kinase JNK, while p38-low cells allow JNK to activate (purple). Finally, all cells that die are from the JNK-high population, while some JNK-high cells and all JNK-low cells survive (black). **(C)** Top: Diagram showing the procession of states and actions for a single cell. Bottom: Black lines follow the actions taken (solid lines) by a single cell out of many possible actions (dashed lines) to transition to new states. The final state of the cancer cell, with the greatest accumulated reward, is continued proliferation. Red lines: By targeting a specific state leading to continued proliferation, we can perturb the cellular rewards to make cell death more favored in cells that would otherwise proliferate.

We present three potential applications where IRL may help uncover key insights for understanding cancer cell heterogeneity. The first two fall into the category of *single-agent IRL*. Here we consider a population of cells observed in our microscopy experiments (e.g., all cancer cells in the field of view) as a collection of single agents operating independently and with no awareness of each other’s actions but obeying the same policy. For a concrete example, consider the behavior of individual cells collected by Miura et al. after exposure to UV-C radiation (Miura et al., 2018). The study identified a molecular determinant of UV-induced cell death by tracking cell motion, kinase activity, and cell survival over time (Figure 2A). Radiation activates JNK kinase after several hours, which induces cell death. Cells that survive radiation first activate p38 kinase, which induces transcription of a regulatory phosphatase that inhibits JNK and prevents cell death.

If we were to use IRL to understand the observations of Miura et al., we could consider JNK, p38, and cell survival/death as key state variables. IRL would first reveal the most common series of events (change in p38, followed by change in JNK, possibly followed by cell death) based on the transitions between states that are present in the data. Identifying the most common state transitions may be trivial in this application, but if more reporters were used or more states were identified from the data, it could be more difficult to identify common series of events. IRL would also show the dominant state-action transitions leading to cell death or survival, where most cells that survive first activate p38. A Sankey diagram (Antar et al., 2022) showing the behavior of 100 hypothetical cells is shown in Figure 2B. Most cells follow the series of events shown in Figure 2A, while a minority do not because of unknown sources of regulation affecting p38 and JNK activity. Miura et al. used separate experiments inspired by biological knowledge to reveal the phosphatase dynamics underlying JNK suppression. Since the phosphatase was not captured in the live-cell imaging experiments, IRL would not be able to identify it. However, IRL would demonstrate that most cells that first activate p38 do not activate JNK, which could generate hypotheses about how these two molecules are connected. Furthermore, after IRL reward inference, we could use the observed rewards to simulate realistic cellular behaviors in different environments or in the presence of different perturbations (Figure 2C). The inferred reward and measured state-action transitions could be used to identify states most likely to lead to cell survival. Identifying these states and targeting them could reveal novel, experimentally testable perturbations to prevent cell survival. In this example, IRL provides a unique, data-driven lens for identifying granular cellular activities that drive specific phenotypes.

As another application, we developed a novel IRL algorithm, called Fokker-Planck IRL (Garikipati et al., 2023), to better understand how chemokine gradients affect cell migration decisions (Ho et al., 2023). FP-IRL infers the transition and reward function simultaneously in a physics-constrained manner by leveraging a mathematical conjecture on a structural isomorphism (i.e., equivalence mapping) between the FP equation, which governs particle motion affected by diffusive and advective forces, and MDP, which is the mathematical basis for IRL. We found that the injection of physical principles mitigates some of the aforementioned challenges, including ill-posedness, physical interpretability, and computational efficiency. We first validated FP-IRL on a synthetic problem that mimics cell migration under a chemotactic gradient. Computational convergence studies showed that FP-IRL can accurately estimate the reward and transition functions we defined in the simulation. To test the method, we then applied FP-IRL to an experimental dataset (1,332 cells over 361 total timesteps) of MDA-MB-231 breast cancer cells expressing fluorescent reporters for Akt and ERK kinases in a chemotaxis assay (Ho et al., 2023). We applied a chemical gradient of chemoattractant CXCL12, which induced cells to move up the gradient. We modeled the cancer cells as decision-making agents under the mathematical formalism of an MDP. We defined x- and y-velocity as state variables and changes in Akt and ERK signaling as actions. FP-IRL identified that cells have a higher reward for migrating up the gradient with relatively high speed, in agreement with our understanding of chemotaxis. Going forward, this method can be applied to understand cell migration strategies in new environments.

Our third potential application employs *multi-agent* IRL to understand competitive and cooperative cellular interactions that support overall tumor progression. Here, we can model each agent (in an overall multi-agent setting) to have its own individual reward function, for instance that might correspond to short-term and long-term goals, or local (agent-level) and global (population-level) goals. Using this approach, we could understand how multiple cancer cells adopt a range of phenotypes (following cell-level rewards) to support the overall proliferation of a tumor (a population-level reward). Experimentally, we could monitor cell proliferation from a small, sparse population of cells to a monolayer, and expose them to sequential doses of different cytotoxic drugs. In this case, we could track the emergence of heterogeneity and the eventual death of part of the population in response to different stressors. By assuming that the dead cells provided some benefit to the living cells and that cell death was state dependent, we could apply multi-agent IRL to understand what state-action pairs had high rewards for the individual cell and which had high rewards for survival of the population as a whole. Multi-agent IRL is much more computationally demanding than traditional, single-agent IRL since it must track and capture the interplay of actions by different agents. New algorithms and methodology are currently under development to overcome the computational challenges.

## Discussion

The framework described in this paper—using IRL together with physically-based mechanistic models to interpret high-dimensional live-cell imaging datasets—has potentially game-changing implications for how we understand and treat cancer. First, it provides a rigorous framework for testing if the hypothesis that cells pursue rewards is relevant to cancer. It is likely true that in some cases, clear rewards can be inferred from heterogeneous cellular behaviors (e.g., cooperation or bet-hedging). However, since cellular regulation is imperfect and generally mediated by local signals, it is also likely that some heterogeneity is random, unregulated, or not driven by cellular cooperation. For behaviors that are reward-driven, we will also learn some of the molecular drivers of cell behavior and potential interventions. Analyzing the reward function will further enable us to develop targeted interventions to control the behavior of cells. By inferring decision-making policies for single-cell and population-scale outputs, we may be able to design therapies to pre-emptively shift cells from aggressive behaviors and disrupt collaborative interactions among subpopulations of cells in a tumor, rather than reacting to these processes after they occur. Combining IRL with physically-based mechanistic models means that we will be able to identify specific, and potentially targetable, drivers of collaborative behaviors.

Although IRL is an emerging technique and questions remain about the application of IRL to single-cell behavioral data, we emphasize that techniques for measuring cell states and actions and achieving granular control over single cells are expanding rapidly. IRL will serve as a powerful method for modeling these new data streams. Specifically, novel reporters have multiplexed up to seven separate fluorescent channels (Qian et al., 2023) and demonstrated the ability to extract single-cell biological information from novel frequency-based fluorescent reporters (Rajasekaran et al., 2024). Such capabilities dramatically expand the range of single-cell states and actions that can be measured. Another emerging approach, where individual cells record a specific physiological variable, such as promoter activity or chemical exposure, onto a protein- (Ravindran et al., 2022; Linghu et al., 2023) or DNA-based (Park et al., 2021) recorder analyzed using endpoint methods, could serve as a novel source for cell state-action data. Finally, recent work has recapitulated fully synthetic kinase networks in mammalian cells (Yang et al., 2023), and optogenetics enables the activation of signaling molecules in cells (Wilson et al., 2017). These tools offer finely tuned control over specific cell behaviors in experimental formats that are compatible with long-term single-cell measurements.

IRL is a general framework that can be adopted for other biological contexts where agent-based perspectives are appropriate. For example, bacteria function as integrated communities, generating interconnected biofilms under stressful conditions. Inflammation in cancer, infections, and other diseases represents a delicate balance between pro-inflammatory and anti-inflammatory cells and molecules. Inferring the cellular reward structure for sustaining or ending inflammation may reveal decision points controlling immunosuppression in tumors and persistent immune responses in autoimmune disorders. We believe IRL will help us understand the underlying causes of cellular heterogeneity by quantifying state-dependent rewards and ultimately contribute to a novel biological paradigm in which the individual roles of heterogeneous cells are considered as the basis of physiological processes.

## Data Availability

The raw data supporting the conclusion of this article will be made available by the authors, without undue reservation.
